# Effectiveness of Aromatherapy Yoga in Stress Reduction and Sleep Quality Improvement among Chinese Female College Students: A Quasi-Experimental Study

**DOI:** 10.3390/healthcare10091686

**Published:** 2022-09-04

**Authors:** Yuan Gao, Jiun-Yi Wang, Fengyi Ke, Rui Tao, Cheng Liu, Shang-Yu Yang

**Affiliations:** 1 Department of Sports, Huazhong Agricultural University, Wuhan 430070, China; 2 Department of Healthcare Administration, College of Medical and Health Science, Asia University, Taichung 41354, Taiwan; 3 Department of Medical Research, China Medical University Hospital, China Medical University, Taichung 404332, Taiwan; 4 College of Basic Medical Sciences, Three Gorges University, Yichang 443002, China; 5 Department of Physical Education, Huazhong University of Science and Technology, Wuhan 430074, China

**Keywords:** aromatherapy yoga, female college students, sleep quality, stress

## Abstract

College students, particularly female students, often suffer from severe stress and poor sleep. Aromatherapy yoga has become a popular exercise in recent years and may help reduce stress and improve sleep quality, although empirical evidence is limited. We investigated the effectiveness of aromatherapy yoga intervention in reducing stress and improving sleep quality among Chinese female college students. A total of 89 participants—44 in the experimental group (aromatherapy yoga) and 45 in the control group (yoga)—with an average age of 19.88 ± 1.13 years, were enrolled in this quasi-experimental study. Interventions were performed in the respective groups once a week for 12 weeks, for 90 min each time. The Wilcoxon signed-rank test and Mann–Whitney U test (including effect size) were used to test the differences in stress reduction and sleep quality before and after the intervention. From the findings of the current research, both therapies did not help female students’ stress levels or sleep hygiene significantly. However, aromatherapy yoga has the potential to improve sleep disturbances experienced by female college students. Additionally, there was no difference in the amount of stress or the quality of sleep between the aromatherapy yoga groups and the regular yoga groups before and after therapy.

## 1. Introduction

College years are considered to be one of the most stressful periods of a person’s life [1]. College students must adjust to a new social environment, a change in lifestyle, increased academic load and must modify their interpersonal relationships [2]. Excessive stress may have negative psychological (e.g., depression, melancholy) and physical (fatigue, muscle tightness) effects [3,4] and may weaken university students’ self-worth, which may affect their studies and even pose a risk of dropping out of college [5,6]. Reportedly, female college students were more likely to be stressed than male college students [7]. Physiological differences between men and women (e.g., gene vulnerability, cortisol levels, and other hormone levels) may manifest via emotions and behaviors [8]. Females and males respond to stress differently because of their differential sensitivity to events [9]. Female university students were found to be more sensitive to pressure than men; hence, they may experience more sadness and anxiety [10]. When stress is not reduced effectively, bad habits [11], such as alcohol consumption [11], smoking [12], and even suicide ideation [13], become common. Additionally, the relationship between stress and sleep quality may be reciprocal. Stress influences sleep quality if it is not dealt with effectively [14,15].

It has been reported that the percentage of Chinese university students with a sleep duration shorter than 7 h per day was 43.9% [16]. The sleep quality of female college students is worse than that of male college students [17]. A study utilizing the Pittsburgh Sleep Quality Index (PSQI) showed that the sleep quality of female participants aged 20–29 years was worse than that of males [18]. Women aged 17–30 years were more likely to have nightmares, delayed sleep onset, and frequent night awakenings [19]. The decrease in sleep quality was probably caused by the hormone cycles of women [20,21]. Female college students with poor sleep quality were more likely to indulge in excessive drinking [22] and self-mutilation [23], which were often accompanied by unhealthy sleep habits such as using mobile phones before sleep [24]. Good sleep quality is a predictor of physical and mental health as well as overall viability [25]. Good sleep quality may contribute to the academic performance of female college students [26] and may help to avoid problems such as adult obesity [27] and drug abuse [28].

Aromatherapy has been demonstrated to be effective in reducing pressure and improving sleep quality [29,30]. Aromatherapy is a complementary and alternative treatment/medicine (CAM), wherein oils extracted from natural sources such as flowers, petals, and bark of plants are used [31] and absorbed via the respiratory system or skin. This is achieved by massage, bathing, and inhalation to improve the physical and psychological conditions of stressed individuals [29,30]. Volatile compounds can be absorbed most rapidly via inhalation, whereby they act through the limbic system, particularly the amygdala and hippocampus [32]. Aromatherapy can reduce sympathetic nerve activity, increase parasympathetic nerve activity, loosen muscles, and adjust the circadian clock, thereby improving sleep quality and relieving a stressed mood [33,34,35]. Compared with drug treatment, aromatherapy is milder and has fewer side effects.

Additionally, yoga is a physical activity preferred by many female college students [36]. Yoga acts as a nonpharmacological, self-empowering CAM that has the potential to enhance stress management [37] and sleep quality in college students [38]. Yoga enhances general health via pranayamas (respiration-control practice), asanas (yoga poses), and chanda (meditative methods), which collectively aim to achieve mental serenity and foster concentration. Asanas and pranayamas can enhance muscular strength and body flexibility and promote blood circulation and hormone function [39]. Meditation is effective in stabilizing the autonomic nervous system, with emphasis on parasympathetic innervation [40] and an effective reduction in arousal [41].

Since aromatherapy has been shown to have psychological effects on mood improvement, using it to help healthy exercisers avoid feeling awful during a workout can be a viable option [42]. If aromatherapy and yoga are combined, their combined positive effects on health care may be enhanced. Therefore, this study aimed to explore the efficacy of aromatherapy yoga in reducing stress and improving sleep quality among Chinese female college students, with a combination of complementary and alternative therapies designed to alleviate female college students’ negative emotions and improve their overall academic performance and mental health.

## 2. Materials and Methods

### 2.1. Study Design and Participants

This study is a quasi-experimental design that lasted from 1 March 2021 to 1 June 2021. Participants were chosen from a specific university in Wuhan, Hubei Province, and only the female students were included. The purpose of this study was explained to the participants by the research assistant, and the students volunteered to participate in the aromatherapy yoga arm (experimental group) or yoga arm (control group) after providing their consent for the study. Both groups attended a 90 min aromatherapy yoga or yoga course once a week for 12 weeks. A questionnaire was administered 1 day before the interventions and included basic information, the Perceived Stress Scale 14 (PSS-14), and PSQI. We included female college students aged > 18 years who were able to understand the questionnaire. We excluded male students, those with a history of respiratory disease, asthma, or allergy to flowers, plants, or essential oils, those who worked at night, and those with a history of mental diseases. The study was approved by the Ethics Committee of Huazhong Agriculture University (HZAUHU-2021-0001) and registered in the ClinicalTrials.gov Protocol Registration and Results System (NCT05459025). All the participants signed an informed consent form.

**Figure 1:**
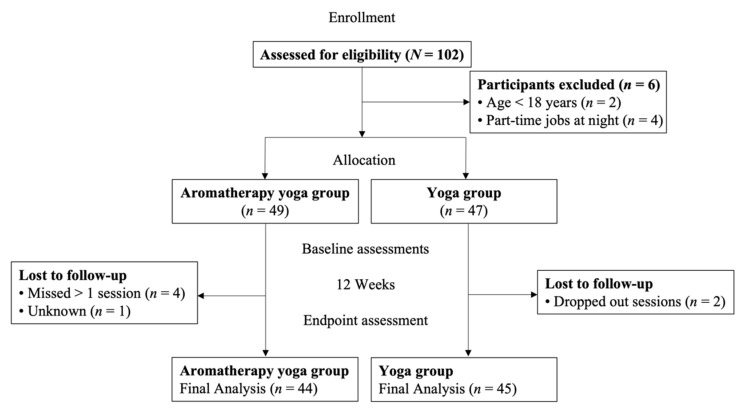
Research flow diagram.

G*Power 3.1. software (Aichach, Germany) was used to calculate the sample size. We used the study by Huang et al. as a reference [43], which had an effect size of 0.74, an alpha level of 0.05, a power of 0.90, and an estimated number of samples of 84. Thus, a total of 102 participants were recruited, and 89 of them completed the study (experimental group, *n* = 44; control group, *n* = 45). Four female college students had part-time jobs in the evening and could not participate in the whole intervention; therefore, they were excluded. A flowchart of this study is shown in Figure 1.

### 2.2. Questionnaire

The first section of the questionnaire included items on basic personal information, such as age, body mass index (BMI), smoking habit (defined as smoking at least one cigarette once a week in the past 6 months), drinking habit (defined as at least three drinks containing alcohol per week in the past 6 months), tea-/coffee-drinking habit (defined as drinking at least three cups of tea/coffee per week in the past 6 months), mobile phone-use habit before sleep (defined as using a mobile phone within half an hour before sleep in the past 1 month).

The second section was the PSS-14 developed by Cohen et al. [44]. PSS-14 is the questionnaire that is most widely used for measuring the degree to which situations in one’s life are appraised as being stressful. The PSS-14 is a 14-item questionnaire, with each item scored on a 5-point Likert scale, ranging from 0 (“never”) to 4 (“very often”). Seven positive and seven negative items were included in the analysis. The total score ranged from 0 to 56 points, with a higher score representing a higher level of perceived stress. The Chinese version of the PSS-14 has good reliability and validity [45]. The Cronbach’s α values for the PSS-14 before and after the study were 0.82 and 0.85, respectively.

The third section was the PSQI developed by Buysse et al. [46], which evaluates the sleep quality of the participants in the past month. This scale is seven subscales (18 items) self-report questionnaire, which includes: (1) Subjective sleep quality (1 item): degree of self-satisfaction in the past month. A higher score represents a more unsatisfactory result; (2) Sleep latency (2 items): the amount of time it took to fall asleep in the past month. A higher score represents more time needed to fall asleep; (3) Sleep duration (1 item): the average sleep time every night in the past month. A higher score represents a shorter sleep time; (4) Habitual sleep efficiency (2 items): higher scores represent lower efficiency of sleep; (5) Sleep disturbances (9 items): higher scores represent more severe sleep disturbances; (6) Use of sleeping medication (1 item): a higher score indicates more frequent use of sleep medication; (7) Daytime dysfunction (2 items): a higher score represents more problems encountered with daily life activities. The score ranges from 0 to 3. The total score ranges from 0 to 21, with higher scores representing worse sleep quality [46]. The Chinese version of the PSQI has good reliability and validity [47]. The Cronbach’s α values before and after the study for the PSQI (including subscales) were above 0.71. 

### 2.3. Intervention

#### 2.3.1. Aromatherapy

Lavender (*Lavandula angustifolia*, of the family *Lamiaceae*) essential oil is a commonly used essential oil that is mild and safe for oral administration [48]. It is not only beneficial for reducing stress and nervousness but also for ameliorating sleep disorders [49]. Therefore, 100% pure *Lavandula angustifolia* essential oil (Erbamea, Italy) was selected, and aromatherapy was implemented by aroma diffusers (W701, fog output volume: 10 ± 4 mL/h, Media).

#### 2.3.2. Yoga

The design of the yoga lessons was based on a past study [43] and a professional yoga instruction book [50]. Yoga lessons were led by certified yoga tutors, with the curriculum contents stated as follows: First, the participant holds her breath and adjusts respiration for approximately 15 min. Subsequently, the participants switched from diaphragmatic respiration to abdominal respiration. Breathing duration was extended progressively to promote both mental and physical serenity, after which the next phase was initiated. The overall duration of the asana exercise was approximately 1 h. It began with a sun-salutation posture as a warm-up, before various asanas, such as the cobra posture, downward facing dog, warrior posture, triangle posture, boat posture, cow-face posture, hero posture 1, hero posture 2, twisted triangle posture, spinal twist posture, seated angle posture, child’s posture, fish posture, wheel posture, locust posture, camel posture, shoulder stand, plow posture, and, lastly, the corpse posture were performed. Eventually, participants spent 15 min in meditation and adjusted their respiration. 

#### 2.3.3. Aromatherapy Yoga

The research assistant diluted the *Lavandula angustifolia* essential oil with distilled water (1:75) 10 min before the yoga session started. The solution was then atomized by three aroma diffusers, placed in the front, middle, and back of the classroom. The distance from each participant was less than 10 m to ensure that the aroma was absorbed completely. The yoga course for the aromatherapy yoga group was the same as that of the yoga-only group. The yoga-only group only took a yoga course without aromatherapy.

Each intervention session in both the study and control groups lasted 90 min, between 6 p.m. and 7:30 p.m. The course was held in a yoga classroom once a week, in an area of 30 m × 35 m. Furthermore, the total intervention period lasted for 12 weeks. The sessions for both groups were scheduled (spread out) to avoid the influence of aromatherapy on the control group. The windows of the classroom were closed during each session. The temperature was maintained between 25 °C and 26 °C, and the average humidity was controlled at 55 ± 5%. The participants were allowed to withdraw if they were uncomfortable. The aromatherapy yoga course was designed by a yoga teacher and a certified aroma therapist. The two groups shared the same yoga teacher.

### 2.4. Statistical Analysis

First, descriptive, statistical demographic data included age, BMI, grade, smoking habit, drinking habit, mobile phone-use habit before sleep, PSS-14 baseline, PSQI total score, and seven subscale scores at baseline. The Wilcoxon rank-sum test and chi-square tests were used to evaluate the differences in demographic data between groups. Second, the Wilcoxon signed-rank test was used to evaluate the within-group difference in the total score of the PSS-14 and the scores of the seven PSQI subscales before and after the study. This method was used because the data were not normally distributed (based on the Shapiro–Wilk test, skewness values for the PSS-14 score, and PSQI subscales ranged from 2.17 to 3.68, whereas kurtosis ones ranged from 2.29 to 11.16). Finally, the Mann–Whitney U test was used to compare the changes (i.e., the difference between the end of the study and the baseline values) of the total PSS-14 score and the scores of the seven PSQI subscales of the two groups to evaluate the efficacy of aromatherapy yoga. The effect size was presented to reflect the difference between the two groups. Effect sizes were calculated using the Mann–Whitney U test (Effect Size r), which captures the standardized median difference between the two groups. Cohen’s classification of effect sizes was used: 0.1 (small effect), 0.3 (moderate effect), and 0.5 and above (large effect) [51]. All calculations were performed using SPSS 25 for Mac (IBM Corp., Armonk, NY, USA). Statistical significance was set at *p* < 0.05.

## 3. Results

Table 1 shows basic demographic information and a comparison of baseline values (PSS-14, PSQI total score with seven scales). There were 89 participants (44 in the aromatherapy yoga group and 45 in the yoga-only group) with an average age of 19.88 ± 1.13 and an average BMI of 20.50 ± 3.38 kg/m^2^. Most participants were freshmen (51.7%), 95.5% of them had no alcohol-drinking habits, and 66.3% had no tea-/coffee-drinking habits. None of the participants had smoking habits, but all had the habit of using cell phones half an hour before bedtime, and all lived in on-campus dormitories. There were no significant differences between the two groups in terms of BMI, alcohol-drinking habits, tea-/coffee-drinking habits, and baseline PSS-14 (*p* > 0.05); however, there were significant differences in age (*p* < 0.001).

Table 2 shows the differences in the PSS-14 and PSQI scores measured before and after the test in the aromatherapy yoga group and the yoga-only group. There was no significant difference within the aromatherapy yoga group in terms of the PSS-14; however, there was a significant difference (*p* < 0.05) in the PSQI subscale of sleep disturbance. This implies that there was a significant improvement in sleep disturbance with the aromatherapy yoga intervention. However, there was no significant difference in the PSS-14, PSQI total score, and seven PSQI subscales in the yoga-only group.

Table 3 displays the differences in PSS-14 and PSQI scores between the aromatherapy yoga and yoga-only group. The PSS-14 and PSQI scores between the two groups were not significantly different. In addition, the effect sizes of sleep length (r = 0.12) and sleep disturbance (r = 0.17) revealed a slight difference between the two groups.

## 4. Discussion

Few studies have examined the combination of aromatherapy and yoga [42] to determine its effectiveness on stress reduction and sleep quality among Chinese female college students. The results of the present study showed that aromatherapy yoga could effectively reduce sleep disturbance in female college students. In aromatherapy yoga and yoga-only groups, there were no differences in stress levels or sleep quality between before and after therapy (except on one PSQI subscale of sleep disturbance in the aromatherapy yoga group).

### 4.1. Stress

As shown in Table 2, there was no significant difference between the aromatherapy yoga and yoga-only groups in terms of stress. Although there have been very few studies on the effects of aromatherapy yoga on an individual’s stress, recent studies have reported the use of lavender essential oil in inhalation aromatherapy to reduce stress levels in female college students, particularly among those who prefer a lavender aroma [52]. Its positive effect on stress levels in different groups, such as women with postpartum stress [53], individuals with academic stress [54], and nurses’ work stress [55], has been demonstrated. Furthermore, it has been reported that performing yoga exercises (lasting 60 min) twice weekly for 7 months rendered a positive influence on female students’ psychoemotional state (i.e., stress), general physical self-feeling, and physical condition [56]. Yoga has also been shown as effective for stress management among healthcare workers [57] and for stress reduction in employees [58]. However, the present study observed a statistically insignificant decrease in PSS-14 scores in both groups after the intervention, but this might have been a result of the study limitations, such as a low intensity of the intervention. Moreover, the baseline score of PSS-14 in this study is lower than those of other college students [58], and thus, there may be less room for change. These could be the reasons the decrease in PSS-14 scores did not reach statistical significance.

In addition, there was no significant difference in the reduction in stress between the aromatherapy yoga and yoga-only groups (Table 3). Both aromatherapy yoga and yoga showed a decreasing trend in PSS-14 scores after the intervention. In other words, both interventions provided some degree of improvement for the participants. This may be one of the reasons for the lack of a significant difference. Further studies should be conducted in this regard in the future.

### 4.2. Sleep Quality

Different groups of people can benefit from the favorable benefits of yoga [59] and aromatherapy [33,60] on their quality of sleep. In this study, the lavender essential oil was used as an intervention. Linalool and linalyl acetate, the two main constituents of lavender oil, have a notable hypnotic effect that promotes sleep by inhibiting the production of acetylcholine and creating a hypnotic effect [61,62,63]. For instance, Gürler et al. [64] discovered that a steam inhalation intervention with a lavender aroma for menopausal women experiencing sleep deficit helped to improve their sleep quality. Additionally, participants saw improvements in subjective sleep quality, sleep latency, sleep length, use of sleep medicines, and particularly sleep disturbance following seven interventions of weekly yoga [65]. The aforementioned factors may help to explain why this study combined yoga and aromatherapy to treat sleep disruption (Table 2). Only the PSQI item measuring sleep disruption showed a significant improvement in the current trial. The usefulness of aromatherapy yoga in enhancing sleep quality has to be further investigated.

There was no significant improvement in sleep quality in the yoga-only group (Table 2), which was inconsistent with previous studies [66,67] related to the effectiveness of yoga in improving sleep quality. Previous studies have noted that low levels of physical activity (e.g., yoga) had no significant effect on improving sleep quality [68,69]. Furthermore, one study noted that general exercise (e.g., yoga) had no significant effect on sleep quality across genders, which could be attributed to relevant demographic variables that were not investigated [70]. Therefore, the lack of a significant effect of the yoga intervention on sleep quality in this study may be due to the low level of physical activity, as well as the uninvestigated demographic variables (e.g., work status and family type), which may have led to the lack of significant difference in the study results. Moreover, the baseline score of PSQI in this study is lower than those of other college students [58], and thus, there may be less room for change.

In Table 3, the differences between baseline and endpoint scores of the aromatherapy yoga group and yoga-only group show that the scores of four components (sleep duration, habitual sleep efficiency, use of sleep medication, and daytime dysfunction) in the aromatherapy yoga group increased, while those of six components of yoga-only group increased, suggesting that most of the PSQI scores were slightly but not significantly increased from the baseline to endpoint. According to the university calendar, the midterm exams were held in the week of the study endpoint. Thus, exam preparation could be a reason students had reduced sleep quality.

In addition, the results in Table 3 show no significant difference in sleep quality improvement between the aromatherapy yoga and yoga-only groups. Considering the relatively small sample size of this study, the statistical test may not have been able to detect significant differences between the two groups because of insufficient test power. The differences in each variable between the two groups are presented in terms of the effect size. Sleep duration and sleep disturbance showed small effect sizes, which suggests a potential better effect of aromatherapy yoga intervention. A previous study showed that the sleep quality of those who were admitted to the intensive care units for percutaneous coronary interventions and underwent aromatherapy was significantly improved compared with those who underwent conventional nursing intervention [71]. Another study showed that a yoga-only group reported significantly lower sleep disturbance scores than the control group, indicating that such a program significantly improves sleep-related outcomes [65]. Therefore, the combination of these two interventions in this study may have reduced sleep disturbance through synergistic effects. The results support the hypothesis that the combination of aromatherapy and yoga may be an effective intervention for reducing sleep disturbance among Chinese female college students. Nonetheless, further studies are required to provide more evidence.

### 4.3. Limitations

This study had some limitations. First, there were significant differences in age between the two study groups at baseline, probably due to nonrandomized allocation into the aromatherapy yoga group since the participants were assigned according to their preference. A randomized study is recommended in the future to reduce bias and extend the sample size to explain causation. Second, response bias could have been induced by the sleep quality scales, although they are extensively used and have good reliability. Therefore, the experimental method may need to consider an objective index to monitor sleep, such as sleep time, sleep cycles, and heart rate variability, in order to reduce the influence of subjective factors. Third, although we utilized G*Power 3.1. software (Aichach, Germany) to calculate the sample size in the initial study stage, more samples are recommended to investigate the efficacy of these two therapies. Finally, sleep disorders are influenced by a variety of other factors, such as the learning pressure in different phases, learning pressure with different majors, and the influence of other factors prior to sleeping. Therefore, future research is needed to consider more compounding factors. In order to control the stability of the intervention effects, it is also advised that future research adopt a nonintervention/aromatherapy-only group as a control group and use three-time measurements: before (Time 1), after therapy (Time 2), and one month/few weeks after therapy (Time 3). Despite these limitations, the findings of this study provide important clues for exploring the efficacy of aromatherapy yoga in improving individual stress and sleep quality.

## 5. Conclusions

Our study suggests that aromatherapy yoga interventions might improve sleep disturbance in female college students. However, the efficacy of improving sleep quality was small compared with that of yoga alone. In general, the therapies did not help female students’ stress levels or sleep hygiene. Therefore, more empirical studies that investigate the effect of aromatherapy yoga intervention on stress and sleep quality in female college students are needed.

## Figures and Tables

**Table 1:** Basic demographic information and comparison of baseline values (PSS-14 and PSQI total score with seven subscales, N = 89).

	Total (*n* = 89)	Aromatherapy Yoga Group (*n* = 44)	Yoga-Only Group (*n* = 45)	t/Z	*p*
**Age**	19.88 ± 1.13	19.32 ± 1.03	18.73 ± 0.91	2.83	<0.001
**BMI (kg/m^2^)**	20.50 ± 3.38	20.32 ± 3.76	20.68 ± 3.00	−0.50	0.63
**Alcohol-Drinking Habit (*n*, %) ^a^**				1.04	0.30
No	85 (95.5)	41 (93.2)	44 (97.8)		
Yes	4 (4.5)	3 (6.8)	1 (2.2)		
**Tea-/coffee-drinking habit (*n*, %) ^a^**				−0.82	0.42
No	59 (66.3)	31 (70.5)	28 (62.2)		
Yes	30 (33.7)	13 (29.5)	17 (37.8)		
**PSS-14**	24.43 ± 6.65	24.22 ± 6.20	24.62 ± 7.13	−0.80	0.43
**PSQI**					
Total score	6.71 ± 2.46	6.86 ± 2.54	6.56 ± 2.40	−0.85	0.40
Subjective sleep quality	1.02 ± 0.64	1.07 ± 0.62	0.98 ± 0.66	−0.72	0.47
Sleep latency	1.44 ± 0.92	1.45 ± 0.87	1.42 ± 0.97	−0.32	0.75
Sleep duration	0.88 ± 0.65	0.89 ± 0.65	0.87 ± 0.66	−0.15	0.88
Habitual sleep efficiency	0.27 ± 0.62	0.32 ± 0.67	0.22 ± 0.56	−0.82	0.42
Sleep disturbance	1.25 ± 0.43	1.30 ± 0.46	1.20 ± 0.40	−1.04	0.30
Use of sleep medication	0.01 ± 0.11	0.02 ± 0.15	0.00 ± 0.00	−1.01	0.31
Daytime dysfunction	1.84 ± 0.69	1.82 ± 0.69	1.87 ± 0.69	−0.47	0.64

^a^ Chi-square test was used; otherwise, Wilcoxon rank-sum test was used. BMI—Body mass index; PSS-14—Perceived Stress Scale 14; PSQI—Pittsburgh Sleep Quality Index; t—*t*-value; Z—*Z*-value.

**Table 2:** Intragroup differences in the PSS-14 and PSQI scores in the aromatherapy yoga group and the yoga-only group.

Components	Aromatherapy Yoga Group				*p* ^a^	Effect Size r	Yoga-Only Group				*p* ^a^	Effect Size r
	Baseline Mean	Baseline Median (IQR)	Endpoint Mean	Endpoint Median (IQR)			Baseline Mean	Baseline Median (IQR)	Endpoint Mean	Endpoint Median (IQR)		
**PSS-14**	24.22 ± 6.20	23.0 (20.0–28.0)	23.45 ± 6.28	22.5 (19.0–28.0)	0.72	0.15	24.62 ± 7.13	25.0 (21.0–28.50)	22.76 ± 8.11	23.0 (16.5–28.50)	0.20	0.19
**PSQI**												
Total score	6.86 ± 2.54	6.5 (6.0–8.75)	6.66 ± 2.69	6.0 (5.0–9.0)	0.98	0.00	6.56 ± 2.40	6.0 (5.0–8.0)	6.98 ± 2.80	6.0 (5.0–9.0)	0.56	0.01
Subjective sleep quality	1.07 ± 0.62	1.0 (1.0–1.0)	1.02 ± 0.59	1.0 (1.0–1.0)	0.69	0.06	0.98 ± 0.66	1.0 (1.0–1.0)	1.02 ± 0.75	1.0 (1.0–1.0)	0.84	0.03
Sleep latency	1.45 ± 0.87	1.0 (1.0–2.0)	1.34 ± 0.81	1.0 (1.0–2.0)	0.62	0.08	1.42 ± 0.97	1.0 (1.0–2.0)	1.27 ± 0.89	1.0 (1.0–2.0)	0.21	0.11
Sleep duration	0.89 ± 0.65	1.0 (0.0–1.0)	0.91 ± 0.77	1.0 (0.0–1.75)	0.94	0.01	0.87 ± 0.66	1.0 (0.0–1.0)	1.16 ± 0.82	1.0 (1.0–2.0)	0.06	0.28
Habitual sleep efficiency	0.32 ± 0.67	0.0 (0.0–0.0)	0.39 ± 0.75	0.0 (0.0–1.0)	0.66	0.07	0.22 ± 0.56	0.0 (0.0–0.0)	0.36 ± 0.65	0.0 (0.0–1.0)	0.24	0.18
Sleep disturbance	1.30 ± 0.46	1.0 (1.0–2.0)	1.07 ± 0.50	1.0 (1.0–1.0)	0.04 *	0.31	1.20 ± 0.40	1.0 (1.0–1.0)	1.18 ± 0.44	1.0 (1.0–1.0)	0.80	0.04
Use of sleep medication	0.02 ± 0.15	0.0 (0.0–0.0)	0.07 ± 0.26	0.0 (0.0–0.0)	0.32	0.15	0.00 ± 0.00	0.0 (0.0–0.0)	0.09 ± 0.47	0.0 (0.0–0.0)	0.18	0.20
Daytime dysfunction	1.82 ± 0.69	2.0 (1.0–2.0)	1.86 ± 0.70	2.0 (2.0–2.0)	0.82	0.03	1.87 ± 0.69	2.0 (1.0–2.0)	1.91 ± 0.82	2.0 (1.0–3.0)	0.69	0.06

^a^ Wilcoxon signed-rank test. * *p* < 0.05. PSS-14—Perceived Stress Scale 14; PSQI—Pittsburgh Sleep Quality Index; IQR—Interquartile Range.

**Table 3:** Intergroup differences in the PSS-14 and PSQI scores in the aromatherapy yoga group and the yoga-only group.

Components	Aromatherapy Yoga Group		Yoga-Only Group		U	Z	*p* ^a^	Effect Size r
	Mean ± SD	Median Value (Interquartile Range)	Mean ± SD	Median Value (Interquartile Range)				
**PSS-14**	−0.77 ± 6.56	0 (−4.25, 2.75)	−1.87 ± 10.79	−3.0 (−6.5, 5)	887.5	−0.84	0.40	0.09
**PSQI**								
Total score	−0.20 ± 3.72	0 (−1.75, 2.75)	0.42 ± 3.91	0 (−2, 2.0)	961.5	−0.24	0.81	0.03
Subjective sleep quality	−0.05 ± 0.86	0 (0, 0)	0.04 ± 1.11	0 (−1, 1)	973.5	−0.15	0.88	0.02
Sleep latency	−0.11 ± 1.22	0 (−1, 1)	−0.16 ± 1.53	0 (−1, 1)	982.0	−0.07	0.95	0.01
Sleep duration	0.02 ± 1.00	0 (−0.75, 1)	0.29 ± 0.97	0 (0, 1)	863.5	−1.10	0.27	0.12
Habitual sleep efficiency	0.07 ± 1.02	0 (0, 0)	0.13 ± 0.76	0 (0, 0)	971.5	−0.18	0.86	0.02
Sleep disturbance	−0.23 ± 0.71	0 (−1, 0)	−0.02 ± 0.58	0 (0, 0)	818.5	−1.57	0.12	0.17
Use of sleep medication	0.05 ± 0.30	0 (0, 0)	0.09 ± 0.47	0 (0, 0)	989.5	−0.01	0.99	0.00
Daytime dysfunction	0.05 ± 1.03	0 (−1, 1)	0.04 ± 1.24	0 (−1, 1)	972.5	−0.15	0.88	0.02

^a^ Mann—Whitney U Test. PSS-14—Perceived Stress Scale 14; PSQI—Pittsburg Sleep Quality Index.

## Data Availability

The data that support the findings of this study are available on request from the corresponding author. The data are not publicly available due to privacy or ethical restrictions.
